# Supplementary material for: Preterm infants’ first breastfeeding attempt: Early initiation and performance: A large multicentre questionnaire study based on maternal observations
Source: PLoS One. 2025 Jul 18;20(7):e0303224. doi: 10.1371/journal.pone.0303224 (PMC12273985; doi:10.1371/journal.pone.0303224)

# Supporting information.

# Preterm infants’ first breastfeeding attempt: Early initiation and performance. A large multicentre questionnaire study based on maternal observations.

S3 Table. Infants' best breastfeeding performance at first attempt across postmenstrual age groups.


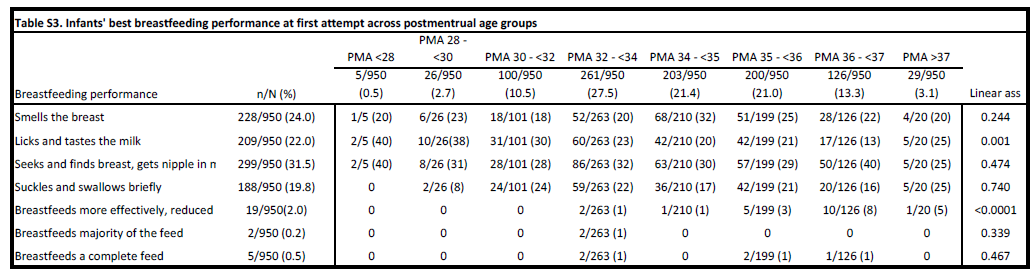

Supplement: S3 Table — (DOCX) [file pone.0303224.s003.docx]
