# Supplementary material for: Preterm infants’ first breastfeeding attempt: Early initiation and performance: A large multicentre questionnaire study based on maternal observations
Source: PLoS One. 2025 Jul 18;20(7):e0303224. doi: 10.1371/journal.pone.0303224 (PMC12273985; doi:10.1371/journal.pone.0303224)

# Supporting information.

# Preterm infants’ first breastfeeding attempt: Early initiation and performance. A large multicentre questionnaire study based on maternal observations.

S4 Figure. Scatter Plot of postmenstrual age at first breastfeeding attempt by gestational age.


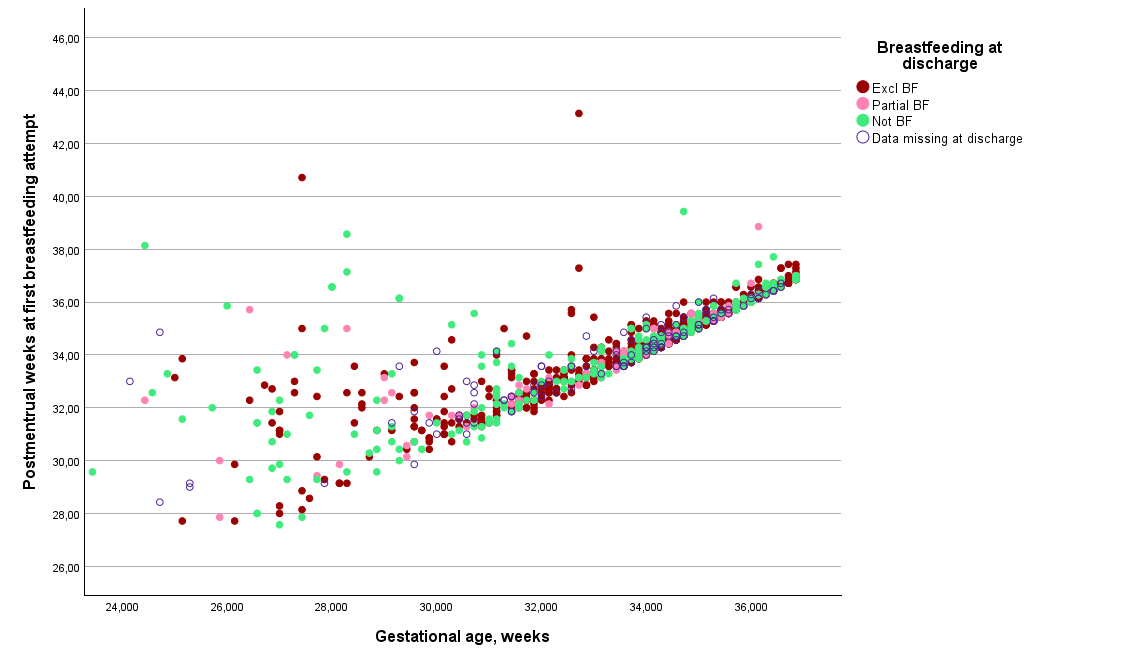

Supplement: S4 Fig — (DOCX) [file pone.0303224.s004.docx]
