# Supplementary material for: Preterm infants’ first breastfeeding attempt: Early initiation and performance: A large multicentre questionnaire study based on maternal observations
Source: PLoS One. 2025 Jul 18;20(7):e0303224. doi: 10.1371/journal.pone.0303224 (PMC12273985; doi:10.1371/journal.pone.0303224)

# Supporting information.

# Preterm infants’ first breastfeeding attempt: Early initiation and performance. A large multicentre questionnaire study based on maternal observations.

S5 Table. Mean differences in days of postmenstrual age at first breastfeeding attempt between infants who did and did not establish exclusive breastfeeding.


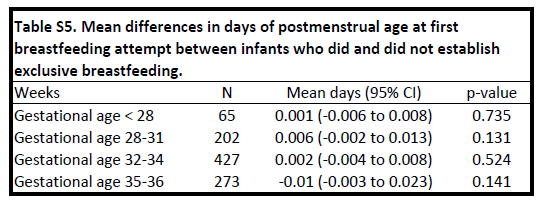

Supplement: S5 Table — (DOCX) [file pone.0303224.s005.docx]
