# Supplementary material for: Preterm infants’ first breastfeeding attempt: Early initiation and performance: A large multicentre questionnaire study based on maternal observations
Source: PLoS One. 2025 Jul 18;20(7):e0303224. doi: 10.1371/journal.pone.0303224 (PMC12273985; doi:10.1371/journal.pone.0303224)
Supplement: Attachment — Submitted filename: Reveiwer report 26-May.docx [file pone.0303224.s006.docx]

**Reviewer Report** Preterm infants’ first breastfeeding attempt: Early initiation and performance with or without nasal-CPAP. A large cohort study

This study is a well-written. It addresses an important issue concerning Early initiation and performance of breastfeeding with or without nasal CPAP

**The title** is adequately addressed.

**There are some comments to help improve the manuscript.**

**Abstract:**

- The abstract provides a brief overview of the study objectives, methods, and key findings. However, it would benefit from including key results with a p-value, which would help the reader understand and make a clearer statement of the study's significance. Additionally, the abstract could mention the potential implications of the findings for clinical practice or patient outcomes.
- Please **check** the manuscript for any grammar mistakes. Some grammar errors require correction, such as
- In line 19 (it should be Baby **F**riendly instead of Baby- friendly)
- In line 40 (put coma**,** after (in 2020)

**Main manuscript:**

- **The introduction** provides a comprehensive background on Early initiation and the importance of breastfeeding in preterm infants with or without nasal-CPAP. The gap is clearly addressed.
- However, it would be helpful to organize the introduction's hierarchical structure and flow and provide contrasting evidence or practices that highlight the research gap or problem your study aims to address.

________

**Methods:** Study design, setting, and participants

- Design should be clearly identified as a “Retrospective multisite observational cohort study”.
- The study mentioned 13 Danish Neonatal Intensive Care Units, and the second line mentioned all 17 Danish Neonatal Intensive Care Units. It would be helpful to clarify whether those numbers are the same.
- Assuming that you selected 13 out of 17 NICUs, but the methodology does not clarify why only 13 of 17 Danish NICUs participated, it would be better to clarify the reasons for non-participation in order to clarify potential biases or limitations in the sample.
- in the design and setting, lines 95- 107 contain a lot of information about the Danish health care system. It would be better to put this information in the introduction.
- In the data collection, it was mentioned that the questionnaires were adapted from a previous study but did not provide specific information regarding the aspects that were modified or revised. This is essential for evaluating the accuracy and dependability of the instruments employed.
- The data were collected from the mothers of preterm infants regarding breastfeeding attempts… If breastfeeding attempts are solely based on maternal reports, recall bias may exist, especially since some data was collected well after events occurred.

_____

**Results:**

- **In the table 1** …..You’ve reported that the preterm(small for gestational age ) was 182/982, and the total number was 992 .. Clarify whether this reflects a subgroup analysis or if there are missing data points for some infants in these categories.
- It was reported that Nasal-CPAP or high-flow nasal cannula (not MV) and Mechanical Ventilation (MV) with different denominators (876 for both), suggesting that not all of the 992 infants are considered in these categories. Clarify whether this reflects a subgroup analysis or if there are missing data points for some infants in these categories.
- It is mentioned in the table comments that 7.9% were mechanically ventilated, followed by nasal-CPAP in all but three infants. Is that reported in the table ?
- It is reported that the mother's characteristics (e.g., Breastfed before 846, Mode of delivery 848) remain inconsistent. Clarify If these figures are supposed to represent subsets due to missing data, it should be explicitly stated.
- It was mentioned that 43/838 for smoking and educational level 838 ……which is inconsistent between smoking and educational level… clarify if this implies missing data if the total is supposed to be 858.
- **In Table 2** ……..It was reported that the total number across all Gestational age groups included for analysis was (968), but it sounds inconsistent because the sum of infants in all GA groups (65 + 202 + 427 + 273 = 967) totals 967, not 968 as mentioned
- **In Table 3**…..can you check the accumulated percentage of the number of infants At <28 weeks was 66 or 65 ?

________

**Discussion:** provides a comprehensive interpretation of the study findings and relates them to the existing literature. When comparing with other studies, we can focus on the differences in care protocols or the sociocultural context that could potentially impact breastfeeding practices. This would bolster the argument regarding the uniqueness or applicability of your findings.

**The conclusion** provides a concise summary of the study's main findings. However, it could be strengthened by emphasizing the practical implications of the results( how change practice in NICUs where breastfeeding initiation is restricted by a fixed PMA and thus benefit preterm infants and their mothers) and suggesting avenues for future research

**Reference:**

- When possible, include more recent studies; older studies, unless they are justified historically, may not reflect the most recent research or guidelines.
