# Supplementary material for: Preterm infants’ first breastfeeding attempt: Early initiation and performance: A large multicentre questionnaire study based on maternal observations
Source: PLoS One. 2025 Jul 18;20(7):e0303224. doi: 10.1371/journal.pone.0303224 (PMC12273985; doi:10.1371/journal.pone.0303224)
Supplement: Attachment — Submitted filename: Response to reviewers.docx [file pone.0303224.s008.docx]

**Answer to reviewer’s comments**

Manuscript: Preterm infants’ first breastfeeding attempt: Early initiation and performance with or without nasal-CPAP. A large cohort study.

We thank you for the opportunity to respond to the valuable comments and suggestions for our manuscript.

We appreciate the constructive feedback provided, which allow us to clarify important aspects of our study.

Hopefully the manuscript is now acceptable for publication.

Below we have responded to every comment.

All line numbers refer to the version with tracked changes.

| **Reviewer’s comment** | **Authors’ answer** |
| --- | --- |
| **Abstract:**  The abstract provides a brief overview of the study objectives, methods, and key findings. However, it would benefit from including key results with a p-value, which would help the reader understand and make a clearer statement of the study's significance. Additionally, the abstract could mention the potential implications of the findings for clinical practice or patient outcomes. | Thank you for the suggestions. We have added a p-value and an implication in the abstract line 30, 34, and 35:  *“Mechanical ventilation significantly delayed first breastfeeding attempt by seven days (p<0.0001).”*  *“Hence, nasal-CPAP should not be a barrier for breastfeeding initiation.”*  Because of word restrictions, we do not mention the differences (or lack of) between GA-groups in the abstract. |
| Please **check** the manuscript for any grammar mistakes. Some grammar errors require correction, such as  In line 19 (it should be Baby **F**riendly instead of Baby- friendly)  In line 40 (put coma**,** after (in 2020) | Thank you. The grammar errors have now been corrected. However, for many years WHO/UNICEF has spelled it “Baby-friendly Hospital Initiative”. When I was part of developing the NeoBFHI 2009-2015, we consulted WHO and they pointed out, that “friendly” did not start with a capital letter.  All WHO documents/publications about BFHI on this WHO’s homepage spell it Baby-friendly Hospital Initiative”.  <https://www.who.int/teams/nutrition-and-food-safety/food-and-nutrition-actions-in-health-systems/ten-steps-to-successful-breastfeeding>  We will therefore use the WHO/UNICEF way of spelling.  Below is a screen print from this URL:  <https://iris.who.int/bitstream/handle/10665/333686/9789240005648-eng.pdf?sequence=1> |
|  | |
| **Main manuscript:**  **Introduction:** The introduction provides a comprehensive background on Early initiation and the importance of breastfeeding in preterm infants with or without nasal-CPAP. The gap is clearly addressed.  However, it would be helpful to organize the introduction's hierarchical structure and flow and provide contrasting evidence or practices that highlight the research gap or problem your study aims to address. | We have re-organized the structure of the introduction and have provided contrasting evidence line 51 and 52:  *However, many studies from Europe, Australia and the U.S. describe breastfeeding initiation from 32 or 34 weeks PMA…* |
| **Methods:** Study design, setting, and participants  Design should be clearly identified as a “Retrospective multisite observational cohort study”. | As this manuscript is part of a larger study, with some data in future articles being prospective, we have designated the study design as follows in line 102:  *An observational multicenter cohort study with retrospective data…* |
| The study mentioned 13 Danish Neonatal Intensive Care Units, and the second line mentioned all 17 Danish Neonatal Intensive Care Units. It would be helpful to clarify whether those numbers are the same. | First, we mention 17 + one units. We will keep that.  In addition, we have clarified in line 107-109:  *“Of the 18 NICUs, 13 participated in the study. Reasons for not participating were other ongoing breastfeeding studies (three NICUs) and lack of time for breastfeeding research (two NICUs).”* |
| Assuming that you selected 13 out of 17 NICUs, but the methodology does not clarify why only 13 of 17 Danish NICUs participated, it would be better to clarify the reasons for non-participation in order to clarify potential biases or limitations in the sample. | See above. |
| In the design and setting, lines 95- 107 contain a lot of information about the Danish health care system. It would be better to put this information in the introduction. | We respectfully believe that the information regarding the setting should remain in the methods section under "setting," as it does not directly contribute to the study's aim and aligns better with the methodological context. We kindly hope this approach will be acceptable. |
| In the data collection, it was mentioned that the questionnaires were adapted from a previous study but did not provide specific information regarding the aspects that were modified or revised. This is essential for evaluating the accuracy and dependability of the instruments employed. | Thank you for the comment. We have clarified in line 139-141:  *“The revision included adding a question about infant performing at first breastfeeding attempt* and deleting questions about breastfeeding experiences in the mother’s network*.”* |
| The data were collected from the mothers of preterm infants regarding breastfeeding attempts… If breastfeeding attempts are solely based on maternal reports, recall bias may exist, especially since some data was collected well after events occurred | Thank you for addressing this, we have however already tried to address this potential bias in the discussion in line 390-395. We hope the changes have made it clearer:  *Information of the first breastfeeding attempt (PMA, performance, and nasal-CPAP) had a maternal recall period of median nine days for 88% and 52 days for 12% of the mothers, respectively. As the first breastfeeding attempt is an important milestone for preterm infants, and since maternal recall of breastfeeding duration has been found valid for a six-year period [39], recall bias should not influence any differences in the analyses of delayed initiation, performance, or nasal-CPAP.* |
| **Results:**  In the table 1 …..You’ve reported that the preterm(small for gestational age ) was 182/982, and the total number was 992 .. Clarify whether this reflects a subgroup analysis or if there are missing data points for some infants in these categories. | Thank you for noticing. The differences are explained by a number of missing data, which have now been clarified. We have now added a column in Table 1, in which the numbers of missing data are presented. Further, we rephrased a sentence in the Statistics line 206-207:  *Some mothers did not answer all of the questions. Mothers and infants with missing data were excluded from the respective analyses.* |
| It was reported that Nasal-CPAP or high-flow nasal cannula (not MV) and Mechanical Ventilation (MV) with different denominators (876 for both), suggesting that not all of the 992 infants are considered in these categories. Clarify whether this reflects a subgroup analysis or if there are missing data points for some infants in these categories.  It was reported that Nasal-CPAP or high-flow nasal cannula (not MV) and Mechanical Ventilation (MV) with different denominators (876 for both), suggesting that not all of the 992 infants are considered in these categories. Clarify whether this reflects a subgroup analysis or if there are missing data points for some infants in these categories. | Thank you for noticing. The differences are explained by a number of missing data, which have now been clarified. We have now added a column in Table 1, in which the numbers of missing data are presented. |
| It is mentioned in the table comments that 7.9% were mechanically ventilated, followed by nasal-CPAP in all but three infants. Is that reported in the table ? | Thank you for noticing. We have now divided the mechanically ventilated infants into two groups in Table 1 (line 214): followed by nasal-CPAP and not followed by nasal-CPAP. |
| It is reported that the mother's characteristics (e.g., Breastfed before 846, Mode of delivery 848) remain inconsistent. Clarify If these figures are supposed to represent subsets due to missing data, it should be explicitly stated. | See above  We have now added a column in Table 1, in which the numbers of missing data are presented. |
| It was mentioned that 43/838 for smoking and educational level 838 ……which is inconsistent between smoking and educational level… clarify if this implies missing data if the total is supposed to be 858. | See above  We have now added a column in Table 1, in which the numbers of missing data are presented. |
| In Table 2 ……..It was reported that the total number across all Gestational age groups included for analysis was (968), but it sounds inconsistent because the sum of infants in all GA groups (65 + 202 + 427 + 273 = 967) totals 967, not 968 as mentioned | Thank you for finding this mistake.  The correct number is 967 and it has now been corrected in table 2 (line 230). |
| In Table 3…..can you check the accumulated percentage of the number of infants At <28 weeks was 66 or 65 ? | Thank you for finding this mistake.  The correct number is 65 and it has now been corrected in table 2 (line 230). |
| **Discussion:** provides a comprehensive interpretation of the study findings and relates them to the existing literature. When comparing with other studies, we can focus on the differences in care protocols or the sociocultural context that could potentially impact breastfeeding practices. This would bolster the argument regarding the uniqueness or applicability of your findings. | Thank you for the suggestions.  We have added to the discussion:  Line 299-301:  *The differences could be explained by local or national care protocols restricting early breastfeeding initiation in some of these countries [14, 15].*  Line 306-309:  *This highlights the need to revise local and national guidelines to incorporate recommendations from the Neo-BFHI and WHO, suggesting that stable preterm infants should have unrestricted access to breastfeeding, regardless of GA, PMA, PNA or current weight [1-3]. Implementation should include training of NICU nurses in readiness for early breastfeeding.* |
| **The conclusion** provides a concise summary of the study's main findings. However, it could be strengthened by emphasizing the practical implications of the results (how change practice in NICUs where breastfeeding initiation is restricted by a fixed PMA and thus benefit preterm infants and their mothers) and suggesting avenues for future research. | The last part of the conclusion has been revised in line 415-420:  *Guidelines and policies in NICUs should comply with the recommendations by Neo-BFHI and WHO. The results can hopefully change practice in NICUs where breastfeeding initiation is restricted by a fixed PMA and thus benefit preterm infants and their mothers by allowing more time for breastfeeding opportunities. Future research should include preterm infants’ physical reactions during first breastfeed attempt with and without nasal-CPAP and their breastfeeding development through the Nine Stages skin-to-skin.* |
| **Reference:**  When possible, include more recent studies; older studies, unless they are justified historically, may not reflect the most recent research or guidelines. | Thank you for the comment. We have carefully gone through all references and deleted the old where they could be replaced with newer references.  Preterm infants breastfeeding performance and breastfeeding with nasal-CPAP are sparsely described and we therefore have to refer to older studies for these topics. |
