# Supplementary material for: Preterm infants’ first breastfeeding attempt: Early initiation and performance: A large multicentre questionnaire study based on maternal observations
Source: PLoS One. 2025 Jul 18;20(7):e0303224. doi: 10.1371/journal.pone.0303224 (PMC12273985; doi:10.1371/journal.pone.0303224)
Supplement: Attachment — Submitted filename: Response to the reviewers second resubmission_2025.01.07.docx [file pone.0303224.s009.docx]

PONE-D-24-15661R1

Preterm infants’ first breastfeeding attempt: Early initiation and performance with or without nasal-CPAP. A large cohort study.

Response to reviewers

| **Reviewers’ comments** | **Authors’ response** |
| --- | --- |
| Reviewer #2: Thank you very much for inviting me to review this article. I read with interest the article by Ragnhild Maastrup and colleagues which analyzed a questionnaire, adapted from their previous study, regarding the performance of preterm infants during breast feeding.  The authors aimed to analyze their previous questionnaire from a different perspective focusing on PMA at first attempt of breast-feeding, factors delaying the first attempt, performance at first attempt with and without nasal-CPAP, and the correlation between PMA at first attempt and PMA at exclusive breastfeeding establishment.  However, the study did not answer the raised questions by the authors as it mainly depended on maternal responses on the performance of their infants during feeding (which is very subjective) rather than a truly structured objective assessment. The data presented are mainly descriptive and lacked a comparative analysis between infants who had early initiation versus those who had late initiation to better understand the obstacles and appreciate the outcomes. | Thank you very much for taking your time to review our manuscript and give useful comments for the improvement of the manuscript. We hope you find that we have addressed all your comments adequately.  With deep respect to you review, we do not agree that mothers’ observations of breastfeeding are invalid. According to fundamental studies by Kersten Hedberg Nyqvist in 1999, mothers’ observations of infant breastfeeding performance are valid. We have stated in the limitation section line 399-401:  *Data on infant performance at first breastfeeding attempt was solely assessed by the mothers. However, Nyqvist et al. found a high interrater reliability between nurses and mothers’ observations of preterm infants breastfeeding performance [4], which justifies why mothers’ observations are considered valid.*  We believe that our study was not designed to a comparative analysis and have stated in the limitation section line 418-420:  *The* *criterium for breastfeeding initiation in all Danish NICUs was “stable infant” and not a certain PMA or weight. Thus, the present study was not designed to determine whether stable infants restricted from early breastfeeding initiation are delayed in timing of establishment of exclusive breastfeeding.*  4.Nyqvist KH, Sjödén PO, Ewald U. The development of preterm infants' breastfeeding behavior. Early Hum Dev. 1999 Jul;55(3):247-64. doi: 10.1016/s0378-3782(99)00025-0. |
| The following points need to be addressed by the authors.  1. The title (Preterm infants’ first breastfeeding attempt: Early initiation and performance with or without nasal-CPAP. A large cohort study) doesn't reflect the design or the methodology of the study. It gives the impression that the design is a comparative analysis between infants initiating breast feeding while on n-CPAP versus those without n-CPAP. It doesn't mention that this is a questionnaire study or a retrospective design. Please follow STROBE stands for reporting observational trials. | Thank you for the suggestion.  We have now changed the title as follows, including more details of the design of the study:  *Preterm infants’ first breastfeeding attempt: Early initiation and performance with or without nasal-CPAP. A multicenter maternal questionnaire study.* |
| 2. What was the criteria for initiation of breast-feeding attempt? Is there a policy for that? Did all the units participated in the study follow the same policy for initiation of attempts? | Thank you for pointing out that the specific criteria for breastfeeding initiation was not clear in the method section. We have added the following, line 109-114:  *Common practice in Danish NICU is that infant stability is the criterion for early breastfeeding initiation, thus following the Neo-BFHI and WHO recommendation, that stable preterm infants should be offered unrestricted access to the breast regardless of GA, PMA, PNA, or current weight [1,2]. NICUs in Denmark comply with the Neo-BFHI definition of infant stability related to breastfeeding: “Infants who respond to routine care and handling without experiencing severe apnoea, desaturation and bradycardia” [1].* |
| 3. If 551 preterm infants were on n-CPAP or HFNC, and 292 (53%) of them received first breast feeding attempt while on CPAP. What controlled infant selection and feeding practice in the participating units? | Thank you for giving us the chance to elaborate on this. All preterm Infants were eligible for inclusion. Breastfeeding with nasal-CPAP is an increasing practice in the participating NICUs, but feeding practice is also depended on the individual nurse.  We have now added more details in the method section line 114-118:  *Even if nasal-CPAP is not an obstacle for breastfeeding initiation, the practice is under evolution in Denmark. For those infants only treated with nasal-CPAP for a few hours, feeding cues might not appear when the mother is present. Further, some of the extremely end very preterm infants could be stable for days before the first breastfeeding attempt depending on the individual nurse.*    We also added in result-section line 229-231:  *Further, we found significant differences between NICUs from 11.2% to 43.8% of infants maintained on nasal-CPAP during first breastfeeding attempt (p<0.0001).* |
| 4. The question of breast-feeding performance was mentioned twice in questionnaire 1 (question number 20) and in questionnaire 2 (question number 4) with the exact same items in the original study. As stated by the authors, the mother received a link to the first questionnaire approximately one week after delivery, and a link to the second at the infant’s final discharge to home. Which response was counted in these results considering that mothers in the second questionnaire has more experience and are more able to retrospectively interpret her infant performance during first attempt in different way? Was there any difference between mother responses in the first and second questionnaires? | Thank you for noticing this. The repeated question in the second questionnaire also included the option “I have answered this question before” and to clarify we have now added the following, line 148-149:  *If mothers nevertheless answered this question twice, the first response was chosen because of the shortest recall period*. |
| 5. In the original study the questionnaires were sent to two groups of mothers, the control group (before training of the nurses) and the intervention group (after training of the nurses). Was there any difference between the two groups? | Thank you for this question.  We have added “comparable” in line 127.  *The study combined data from two comparable cohorts of preterm mother-infant dyads…*  As early breastfeeding initiation was not part of the nurses’ training in the original study, and as the two groups were comparable, the participants were combined into one group (line ca. 128-130).:  *The two cohorts were part of a pre-post staff training intervention study improving exclusive breastfeeding in NICUs [24]. First breastfeeding attempt was not part of the intervention.*  We can answer your question by a rough analysis: There was no significant difference in mean PMA at first BF attempt between the pre- and post-group in the original study (PMA 34.059 vs 34.063 weeks, p=0.177).  As this manuscript is not aiming for comparing outcomes between the two original groups, we think it is not appropriate to report them as a pre- and post-group or give individual results for the two groups. |
| 6. The first item in the performance at first feeding attempt was "Smells the breast", What was the infant reaction which gave the mother this impression? How was the mother able to conclude that her infant smelled the breast particularly in the group of infants on n-CPAP with the interface on? | Thank you for this clarifying question. We have improved the description in the variable section line 167-168:  ”*Smells the breast” is the step on the Milky Way that might not include activity from the infant and could also be described as “mouth/nose against nipple”.* |
| 7. Is there any chance that the infant did not do any reaction or response while on the breast? This option was not given to the mother in the original questionnaire. What are the chances that mothers have chosen the option of "Smell the breast" when their infants had no response as an alternative response? | Thank you for giving us the chance to clarify this.  As we described in the previous response, we have improved the description in the variable section line 167-168:  *”Smells the breast” is the step on the Milky Way that might not include activity from the infant and could also be described as “mouth/ nose against nipple”.*  Further, in the limitation section (line 407-409) we added:  *It was not possible to answer “no reaction” as a response to performance at the first breastfeeding attempt, but the step ”Smells the breast” could also be interpreted as “mouth/nose against nipple” which might not include reactions from the infant.* |
| 8. Although the authors, were interested in assessing the value of early initiation of breast feeding (PMA at first attempt) on the later ability of full breast feeding (PMA at exclusive breastfeeding establishment), they included 273 (27.5%) infants of 35 to 36 weeks' gestation who have inherited ability to establish breast feeding and will, probably, not benefit from early intervention. I believe this is the reason that the authors did not find a significant correlation between PMA at first breastfeeding attempt and PMA at establishment of exclusive breastfeeding (Pearson's correlation 0.002 (p=0,970). A subgroup correlation analysis for infants less than 32 weeks' gestation in whom early intervention is expected to make a difference may have a different significance. | Thank you for the suggestion. We performed a subgroup analysis of infants less than 32 weeks and found no significant correlation. We have added a scatterplot of PMA at first breastfeeding attempt and PMA at establishment of exclusive breastfeeding as a supplementary figure for better visual understanding. In the result section we added line 296-298:  *as well as no correlation among infants with GA<32 weeks (Pearson’s correlation -0.022 (p=0.812)), see Supporting Information Scatterplot Figure S5.*   |
| 9. The results of Table 2 are very expected and too obvious to be compared statistically. 35- and 36-weeks infants are usually born to the breast directly, what is the value of comparing their PNA and weight at first feeding attempt to a preterm infant less than 28 weeks' gestation? | Thank you for letting us elaborate on this. We agree with the reviewer that late preterm infants will initiate breastfeeding later than PMA 35. The late preterm infants in our study did however not have uninterrupted skin-to-skin contact directly from birth and until first breastfeeding was completed as we only included late preterm infants in need of additional treatment and care and thus, admitted to the NICU (line 104 and 131).  The value of comparing is to show that these infants are not initiating breastfeeding at a fixed PMA.  In countries with a fixed PMA for breastfeeding initiation there might not be differences between extremely, very, and moderate preterm infants’ PMA at first breastfeeding attempt. Our analysis indicates that breastfeeding of preterm infants in our cohort is not initiated at a fixed PMA.  The Chi^2^ test could be redundant, but some readers may speculate if the differences were significant, and therefore we wish to keep this information in the manuscript and we hope the reviewer will accept this choice. |
| 10. Table 4 looked only on gestational age, respiratory support, SGA, and previous maternal experience with breast feeding as factors that may delay initiation of breast feeding. However, I believe there are other many factors which are of importance including (and not limited to) neonatal sepsis, inotropes, PDA and PDA treatment, etc. | Thank you for this relevant comment. We agree with the reviewer that other factors related to the infant’s treatment could be of importance, but since we do not have data on e.g., neonatal sepsis, inotropes, PDA and PDA treatment or other factors, we have not been able to include this information. We do however also believe that the sicker infants have been captured by the variable mechanical ventilation as this is only used for very challenged preterm infants in Denmark.  The following limitation has now been added to the limitation section (line 409-412):  Another limitation is that details on infant treatment that could delay breastfeeding initiation were limited to respiratory support. However, as mechanical ventilation is only used for very challenged preterm infants in Denmark, we do believe that the influence of sicker infants was captured in that variable. |
| 11. Table 5 the number of infants with a response of "Smell the breast" to the total population should be 234/992 and not 243/992. | Thank you very much for finding this type error. It has now been corrected. |
| Reviewer #3: Thank you for asking me to review this revised manuscript. \i think the manuscript is nicely presented. I this there is a few points which needs some clarity.  The first point is related to mechanical ventilation, both direct and indirect ventilation. It is a bit confusing how this data is being presented. in the smaller infants, <28 weeks and 28-31 weeks, it is not clear in those groups who were still on NCAP or HF at the time of the first BF attempt. Also what steps were taken in the NICUs for babies to be given the go ahead with respect to first attempts. Were volumes, as determined by test weighing, logged. This may be difficult to answer as the data is based on maternal questionnaires. | Thank you very much for taking your time to review our manuscript and give useful comments for the improvement of the manuscript. We hope you find that we have addressed all your comments adequately.  We have added a Table S2 in Supporting information that reports proportions of infants maintained on nasal-CPAP at first breastfeeding attempt in each GA group and added the following text in the result section line 227-229:  *The lower the GA group the more infants were maintained on nasal-CPAP at the first breastfeeding attempt from 58/69 (84.1%) in extremely preterm infants to 59/273 (21.6%) in late preterm infants (Linear-by-Linear Association <0.0001, see full table in Supporting information S5).*  Thank you for pointing out that the specific criteria for breastfeeding initiation was not clear in the method section. As also described in comment 2 from reviewer 2, we have now added line 109-118:  *Common practice in Danish NICU is that infant stability is the criterion for early breastfeeding initiation, thus following the Neo-BFHI and WHO recommendation, that stable preterm infants should be offered unrestricted access to the breast regardless of GA, PMA, PNA, or current weight [1,2]. NICUs in Denmark comply with the Neo-BFHI definition of infant stability related to breastfeeding: “Infants who respond to routine care and handling without experiencing severe apnoea, desaturation and bradycardia” [1]. Even if nasal-CPAP is not an obstacle for breastfeeding initiation, the practice is under evolution in Denmark. For those infants only treated with nasal-CPAP for a few hours, feeding cues might not appear when the mother is present. Further, some of the extremely end very preterm infants could be stable for days before the first breastfeeding attempt depending on the individual nurse.* |
| The second point is related to the questionnaires completed by the mothers. There seems to be very large window for completion. Is there an explanation and what steps were in place regarding getting the questionnaires? | Thank you for the opportunity to improve the description of reminders send to the mothers.  As the second questionnaire was answered at the infant’s discharge to home, the most preterm infants had higher postnatal age when the questionnaire was answered. That explains the large window for completion and the long recall period for 12% of the mother-infant dyads (line 232).  We added that the link was sent by e-mail (line 152).  Regarding steps in place for getting the answered questionnaires, we have specified that reminders was send during the following month (line 154-155):  *Reminders were sent alternately by text message and e-mail at least three times in the following month.* |
