# Supplementary material for: Preterm infants’ first breastfeeding attempt: Early initiation and performance: A large multicentre questionnaire study based on maternal observations
Source: PLoS One. 2025 Jul 18;20(7):e0303224. doi: 10.1371/journal.pone.0303224 (PMC12273985; doi:10.1371/journal.pone.0303224)
Supplement: Attachment — Submitted filename: Response to reviewers third re-submission.docx [file pone.0303224.s010.docx]

PONE-D-24-15661R1

Preterm infants’ first breastfeeding attempt: Early initiation and performance. A large multicentre questionnaire study based on maternal observations.

We have responded point by point to the latest comments from reviewer #3.

We added numbers to the comments.

| **Reviewers’ comment** | **Authors’ response**  All line numbers refer to the “track changes version”. |
| --- | --- |
| Reviewer #3: Thank you for asking me to review the revised manuscript. I think that describing the association between first exposure and feeding at final discharge is extremely important. | Thank you for reviewing the manuscript again and giving valuable comments. We are convinced that these comments have improved the manuscript. |
| 1.  I am not sure that you addressed the concerns expressed by the reviewers related to those infants who had their first exposure to the breast while on CPAP. The standard of care for the majority of preterm infants, especially now is to use less invasive ventilation to reduce barotrauma. So it is important to recognize this approach in care and not to minimize lung disease and immaturity. The findings as you describe in Table 1 shows that when babies are born after 30 weeks gestation, they do have the capacity to develop feeding skills but these babies are more mature. What is still not clear is how are babies born under 28 weeks determined to be stable enough to experience exposure. What medical criteria was in place to consider this. This was not addressed and I think that this is a major limitation and should be identified as such. Maternal recall may not be able to address this. | Thank you for possibility to further elaborate on infant stability.  Danish NICUs use the Neo-BFHI definition of stability related to breastfeeding to determine breastfeeding initiation. These criteria are well known and easy to use among Danish neonatal nurses. These criteria were later repeated by WHO.  To make this clearer to the reader, we have now added WHO as reference and added, lines 112-115:  *NICUs in Denmark comply with the WHO and Neo-BFHI definition of infant stability related to breastfeeding: “Infants who respond to routine care and handling without experiencing severe apnoea, desaturation and bradycardia” including lack of significant blood pressure fluctuations [1-3].*  This is what WHO writes about stability in the 2020 document “Protecting, promoting and supporting breastfeeding: the Baby-friendly Hospital Initiative for small, sick and preterm newborns” page 18 (same definition of stability for skin-to-skin contact and for breastfeeding initiation):  “The WHO defines “stable” as the absence of severe apnoea, desaturation and bradycardia *(1)*. In practice, the definitions of “stable” and “stabilized” vary widely, but should not be unreasonably restrictive, should be based on evidence (including expert experience and opinion), and be clearly defined in the neonatal ward skin-to-skin care policy or protocol *(208).* The definition of stable may also include lack of significant blood pressure fluctuations.*“*  And  “Stable infants should be offered unrestricted access to the breast, regardless of gestational age or weight *(211*-*213)*. Mothers or infants who are unstable following delivery may need to delay the initiation of breastfeeding until the mother and infant are deemed stable and can be together. It is for this reason that the BFHI global standard of 80% is not applied to preterm infants. However, as with skin-to-skin, facilities should be encouraged to set both realistic and ambitious targets for their setting. “  WHO refers to its own document from 2018 (Implementation guidance: protecting, promoting and supporting breastfeeding in facilities providing maternity and newborn services – the revised Baby-friendly Hospital Initiative. Geneva: World Health Organization; 2018. Licence: CC BY-NC-SA 3.0 IGO). Here with the quote from page 23 :  “Preterm infants may be able to root, attach to the breast and suckle from as early as 27 weeks’ gestation *(67)*. As long as the infant is stable, with no evidence of severe apnoea, desaturation or bradycardia, preterm infants can start breastfeeding.”  In addition, we have now described it as a limitation in lines 429-435:  *The definition of infant stability related to breastfeeding was “Infants who respond to routine care and handling without experiencing severe apnoea, desaturation and bradycardia” [1-2,3]. This definition could, in practice, vary widely. As stability was not clearly defined in the NICUs’ protocols, it could be interpreted differently between and within participating NICUs. Further, the interpretation of stability could vary depending on the health professionals experience with early breastfeeding and could limit implementation of new practice based on the present results. WHO suggests NICUs not to be unreasonably restrictive and to "set both realistic and ambitious targets” for early breastfeeding in preterm infants [3].*  Thank you for the chance to elaborate on the more mature infants’ capacity to develop feeding skills. We think you are referring to the results in Table 5.  We did not look at the development for each infant, but only at the performance at the very first BF attempt as described in lines 343-344:  *As we did not measure the single infant’s development of breastfeeding competencies over time, we are not able to describe the pace in the development.*  Surprisingly, most infants in all GA-groups performed in the first steps of the Milky Way which could be referred to as familiarization. When it comes to “Breastfeeding more effectively” we found that significantly more infants with GA 32-36 reached that step than those with GA less than 32 weeks (Table 5). And the last two steps were predominantly reached by infants GA 32-36. We have added more details to the results lines 278-279:  *The very few infants (2.6%) who displayed a breastfeeding behaviour beyond this at the first breastfeeding attempt were predominantly moderate and late preterm infants (GA 32 – 36).*  And added more details to the discussion lines 343-345:  *Breastfeeding behaviour at the first breastfeeding attempt beyond familiarization and briefly sucking was almost only seen in infants born from 32 gestational weeks, who are more mature, still this performance was rare.* |
| 2.  It is also important to note that you have a number of mothers who chose not to complete the questionnaire. This needs to be addressed in the paper. You also do not give any idea as to how babies were determined to be stable. That is a limitation because this paper is based strictly on maternal recall. You did not get any data about the babies directly from a medical perspective. From a neonatologist perspective, this is critical especially for the extremely immature preterm infant. | Thank you for the suggestion of improvement om the selection of participants.  The first questionnaire was sent to the mothers approximately one week after delivery. The second at the infants’ discharge. The second questionnaire included data on bottle-feeding as well as breastfeeding at discharge. Some respondents were lost to follow-up, why data was missing.  We added information in line 213-214 (italic):  Some mothers did not answer all of the questions *or did not answer the second* questionnaire *about breastfeeding at discharge.* Mothers and infants with missing data were excluded from the respective analyses.  We added markers for breastfeeding status at discharge including a marker for missing data in Figure S2 and addressed it in the discussion lines 373-375:  *The missing data for 12% of the infants could potentially contribute to the lack of significant differences but missing data seems randomly distributed (Supporting information, Figure S2), and the observed differences very small (Supporting information, Table S5).*  Thank you for the possibility to further clarify on the judgement of infant stability. As mentioned in the response to comment 1, Danish NICUs use the WHO and Neo-BFHI definition of stability. Thus, timing of first breastfeeding attempt for infants below 34 weeks would be on the initiative of the neonatal nurse, not the mothers, and we have no reason not to rely on competencies of the neonatal nurses’ judgement of stability.  Only breastfeeding initiation for late preterm infants without respiratory support could be on the mother’s initiative.  Lines 115-116:  Infant stability will be assessed by the competent neonatal nurses.  We agree that the absent of medical criteria for stability could limit the implementation in settings that are culturally different from ours, and we have now added this sentence to the limitation section lines 423-425: *We had no information of the infants’ medical conditions at first breastfeeding attempt or the physical reactions during the first breastfeeding attempt e.g., desaturations, bradycardia.*  As described above, we further added to the limitation section lines 429-435:  *The definition of infant stability related to breastfeeding was “Infants who respond to routine care and handling without experiencing severe apnoea, desaturation and bradycardia” [1,2]. This definition could, in practice, vary widely. As stability was not clearly defined in the NICUs’ protocols, it could be interpreted differently between and within participating NICUs. Further, the interpretation of stability could vary depending on the health professionals experience with early breastfeeding and could limit implementation of new practice based on the present results. WHO suggests NICUs not to be unreasonably restrictive and to “set both realistic and ambitious targets” for early breastfeeding in preterm infants [3].* |
| 3.  In the paper, you state that 1819 infants were eligible but only 1243 were included; why were 576 not included and what were the reasons for not including them. we should also know what was the distribution of the entire eligible infants and why they ended up being excluded. In total 992, which is just over 50%, were included. From a research perspective, this does not allow adequate information to determine generalizability. | To make this clear, we have now added more details regarding this in lines 218-223:  *In total 1819 preterm infants were eligible. Mothers of 125 infants were particularly vulnerable (e.g., psychiatric problems, extremely stressed by their critically ill infant) and not approached for inclusion, further, the NICUs randomly forgot to include 174 infants. Mothers of 1520 infants were informed and 277 declined to participate. Consent for participation was obtained for 1243 infants and 992 (79.8%) of them had available data on performance at first breastfeeding attempt (see Flowchart, Figure 2).*  We added limitations of generalizability in lines 438-440:  *Some mother-infant pairs were for different reasons not included in the study for whom we had no data. Thus, the results of our study could only be generalized to similar populations.* |
| 4.  Although I recognize the value of maternal input, very little is provided regarding medical stability. This was not addressed in the paper. Consequently little guidance is provided to the reader on how to implement steps for the very preterm infant. This again is a limitation. As authors, you were able to show it is possible but provided little more than that. | As responded to comment 1, we acknowledge that the absent of medical criteria for stability could limit the implementation in settings that are culturally different from ours, and this has been included as a limitation in lines 432-434.  *Further, the interpretation of stability could vary depending on the health professionals experience with early breastfeeding and could limit implementation of new practice based on the present results.*  We have moderated the generalizability and implications in the conclusion:  Added “in this cohort” in line 442:  The majority of the extremely and very preterm infants *in this cohort* had their first breastfeeding attempt before 32 weeks PMA.  Added “*based on WHO’s definition” in l*ine 449:  Hence, nasal-CPAP should not be a barrier to breastfeeding initiation in stable infants *based on WHO’s definition.*  Reformulated lines 452-455:  *Based on our findings, NICUs where breastfeeding initiation is restricted by a fixed PMA may be inspired to follow WHO’s recommendations and set realistic and ambitious targets for early breastfeeding initiation in preterm infants…* |
| 5.  The title is misleading in that it suggests that you evaluated the role of CPAP more in depth which in fact you did not. Consequently, this needs to be addressed and is a limitation and the title needs to change. | We recognize that the title could be misinterpreted, and the title has now been changed:  *Preterm infants’ first breastfeeding attempt: Early initiation and performance. A large multicentre questionnaire study based on maternal observations.* |
|  | In addition, we have reordered the tables and figures in supporting Information |
