# Supplementary material for: Preterm infants’ first breastfeeding attempt: Early initiation and performance: A large multicentre questionnaire study based on maternal observations
Source: PLoS One. 2025 Jul 18;20(7):e0303224. doi: 10.1371/journal.pone.0303224 (PMC12273985; doi:10.1371/journal.pone.0303224)
Supplement: Attachment — Submitted filename: Rresponse to reviewers fourth re-submission.docx [file pone.0303224.s011.docx]

**Answer to reviewer #4**

Review Comments to the Author
Reviewer #4: The authors address an important topic and describe a practice that is not common which is allowing premature babies to attempt breastfeeding at a lower age than developmentally capable and while on respiratory support. In this observational muliticenter cohort study, it was noted that preterm newborns as early as 27 weeks attempted breastfeeding, the main reason to prevent such attempts per this study were patients being on mechanical ventilation. However this practice did not affect the rate of breastfeeding later on.

| Comments from reviewer #4 | Authors’ response |
| --- | --- |
| The authors have addressed the concerns previously raised by reviewers but I would still ask for some clarifications on the following matters: - The study is from the period 2016-2019, which is now 6-9 years old, have their been any changes in practice or recommendations on that matter? Can the authors clarfiy that in the discussion section? | Thank you for the relevant suggestion. We have added line 439-441:  *The study was conducted in 2016 – 2019, and the national recommendation on breastfeeding initiation among stable preterm infant has not changed.* |
| - For the second questionnaire, the recall time was 52 days which increases recall bias especially for mothers describing their first attempt and in order to plot it on the "milky way". | We acknowledge the comment from the reviewer and have already tried to address this in the limitation section line 414-418, where we describe that 12% had a recall period on 52 days (median) and 88% had a period of only nine days (median) of recall:  *Information of the first breastfeeding attempt (PMA, performance, and nasal-CPAP) had a maternal recall period of median nine days for 88% and 52 days for 12% of the mothers, respectively. As the first breastfeeding attempt is an important milestone for preterm infants,* *and since maternal recall of breastfeeding duration has been found valid for a six-year period [42], recall bias should not influence any differences in the analyses of delayed initiation, performance, or nasal-CPAP.*  To further clarify, that in Denmark ‘The Milky Way’ has been used since 2008 to inform parents and health care professionals about breastfeeding among preterm infants, suggesting that the mothers were likely to be familiar with the steps, we have now added the following to the methods section line 168-170:  *The Milky Way has been used nationwide in Denmark since 2008 to inform parents and health care professionals about preterm breastfeeding [26]. Mothers of preterm infants were therefore likely to be familiar with the steps in the Milky Way.*  New reference added: Danish Health Authority 2008. Breastfeeding – a handbook for health care professionals. |
| - The authors mention that babies had to be clnically stable in order to attempt breastfeeding, but there were no adverse events reported during these attempts. Was this monitored or part of the questionnaire? The authors should explicitly mention that, especially that this practice was not associated with successful establishment of exclusive breastfeeding. This would help the reader balance the benefit vs risk of incorporating this practice. | Thank you for the possibility to further clarify.  Adverse events were not monitored or part of the questionnaire, which has been described as a limitation line 424-429:  *We had no information of the infants’ medical conditions at first breastfeeding attempt or the physical reactions during the first breastfeeding attempt e.g., desaturations, bradycardia. In Denmark, severe adverse events are systematically reported to learn and prevent similar situations. Our study was led by the Knowledge Centre for Breastfeeding Infants with Special Needs. No severe adverse events during breastfeeding of preterm infants have been reported to the Knowledge Centre during the study period*.  Further, we have already tried to explicitly mention that we did not find statistically significant associations between PMA at first breastfeeding attempt and exclusive breastfeeding at discharge, in the results section line 302-307:  *There was no correlation between PMA at first breastfeeding attempt and PMA at establishment of exclusive breastfeeding in all infants (Pearson's correlation 0.002 (p=0,970), as well as no correlation among infants with GA<32 weeks (Pearson’s correlation -0.022 (p=0.812)), see Supporting Information Scatterplot Figure S4. Within each GA group there was no association between PMA at first breastfeeding attempt and successful establishment of exclusive breastfeeding (Supporting information, Table S5).*  and in the discussion section line 372-374:  *Moreover, we found no association between PMA at first breastfeeding attempt and exclusive breastfeeding at discharge in this cohort which could be explained by the high level of breastfeeding support to all dyads.*  This is repeated in the conclusion line 452-454: *We found no correlation between PMA at first breastfeeding attempt and PMA at establishment of exclusive breastfeeding, nor exclusive breastfeeding at discharge.*  Infants in this study were not restricted from early breastfeeding initiation. To clarify the limitations of the design we added “*or are less breastfed.*“ in line 439.  Line 436-439: *The criterium for breastfeeding initiation in all Danish NICUs was “stable infant” and not a certain PMA or weight. Thus, the present study was not designed to determine whether stable infants restricted from early breastfeeding initiation are delayed in timing of the establishment of exclusive breastfeeding or are less breastfed.*  We hope the reviewer will find this appropriate. Otherwise please let us know. |
